# Regulatory Bottlenecks Linking HDL Dysfunction, Inflammation, and Oxidative Stress: A Petri Net-Based Systems Analysis

**DOI:** 10.3390/ijms27188066

**Published:** 2026-09-10

**Authors:** Dorota Formanowicz, Adam Aron Rynkiewicz, Piotr Formanowicz

**Affiliations:** 1 Department of Medical Chemistry and Laboratory Medicine, Poznan University of Medical Sciences, Rokietnicka 8, 60-806 Poznan, Poland; 2 Institute of Computing Science, Poznan University of Technology, Piotrowo 2, 61-138 Poznan, Poland; aron.rynkiewicz@cs.put.poznan.pl (A.A.R.); piotr@cs.put.poznan.pl (P.F.); 3 Data Analytics and Semantics Department, Poznan Supercomputing and Networking Center, Jana Pawla II 10, 61-139 Poznan, Poland

**Keywords:** high-density lipoprotein (HDL), inflammation, oxidative stress, immunometabolism, cardiovascular disease, cholesterol efflux, Petri nets, systems biology, regulatory bottlenecks

## Abstract

High-density lipoprotein (HDL) is a multifunctional lipoprotein involved in lipid transport, immune regulation, and redox homeostasis. Chronic inflammation and oxidative stress alter HDL composition and function, promoting the formation of dysfunctional HDL particles, impairing cholesterol efflux, and contributing to cardiovascular disease progression. To investigate the regulatory mechanisms underlying these changes, we developed a Petri net-based model of HDL metabolism and extended it to represent inflammatory conditions and selected therapeutic interventions. As a literature-derived qualitative model, it provides a simplified systems-level representation of HDL metabolism and its interactions with inflammation and oxidative stress. Nevertheless, the model captures the essential processes governing these pathways. Using in silico knock-out analysis, we evaluated the impact of disabling individual processes on global network dynamics and identified key regulatory bottlenecks. Network control was highly uneven and concentrated within a limited set of transitions. Under inflammatory conditions, the dominant bottlenecks included reduced LCAT-mediated cholesterol esterification, adiponectin-dependent regulation, and oxidized HDL-associated activity, indicating that HDL dysfunction arises from a tightly interconnected immunometabolic network linking lipid metabolism, inflammation, and oxidative stress. Therapeutic interventions reorganized network control toward the inhibition of inflammation, the restoration of redox balance, and the reconstitution of HDL-mediated cholesterol export. Inhibition of inflammation and oxidation-reduction remained dominant regulatory nodes across pathological and therapeutic states, suggesting a core regulatory backbone of HDL metabolism. These findings demonstrate that inflammation and oxidative stress fundamentally reshape HDL network control and identify a limited set of regulatory bottlenecks that may represent promising targets for therapeutic intervention in cardiovascular and chronic inflammatory diseases. However, because the model represents a simplified qualitative abstraction of HDL biology, the identified bottlenecks should be interpreted as systems-level hypotheses requiring further experimental validation.

## 1. Introduction

High-density lipoprotein (HDL) plays a crucial role in human physiology. First, HDL is responsible for transporting lipids within the circulatory system, facilitating reverse cholesterol transport and maintaining lipid homeostasis [1]. In addition to this fundamental function, HDL exhibits a wide range of protective biological activities, including anti-inflammatory, anti-diabetic, and anti-thrombotic effects [1,2,3]. These properties underscore its importance in cardiovascular and metabolic health.

HDL is highly heterogeneous in both protein and lipid composition [4]. Consequently, its metabolism is complex and tightly regulated by multiple interconnected factors [5]. Among these, inflammation plays a particularly significant role. Although HDL is generally considered anti-inflammatory, accumulating evidence indicates that its functional properties are strongly dependent on its composition. Alterations in HDL composition can lead to a functional shift from anti-inflammatory to pro-inflammatory behavior [5,6,7,8].

Furthermore, several studies suggest that simply increasing HDL-C levels does not necessarily translate into improved clinical outcomes, highlighting the importance of HDL functionality rather than quantity alone. Recent evidence also indicates that very high HDL-C concentrations may not always confer cardiovascular protection, challenging the traditional view of HDL-C as a uniformly beneficial biomarker [9,10,11,12,13,14]. In addition, inflammation itself can alter HDL metabolism, impair its protective functions, and create a feedback loop that further exacerbates inflammatory processes [15]. These observations point to a highly dynamic and context-dependent interplay between HDL and inflammation.

Despite extensive research on HDL biology, most studies have traditionally focused on isolated pathways or single molecular components, often overlooking the complex network of interactions that govern HDL metabolism in vivo. However, growing evidence suggests that HDL functionality is determined not solely by its concentration but also by its dynamic remodeling, composition, and interactions with metabolic and inflammatory processes.

Inflammation introduces coordinated perturbations across multiple pathways, including cholesterol efflux, lipoprotein remodeling, and oxidative balance, leading to a shift in HDL function from atheroprotective to dysfunctional states.

These observations highlight the need to move beyond a reductionist perspective and adopt a systems-level framework, in which HDL metabolism is understood as a multiorgan, interconnected network encompassing the liver, intestine, peripheral tissues, and immune cells.

**Figure 1:**
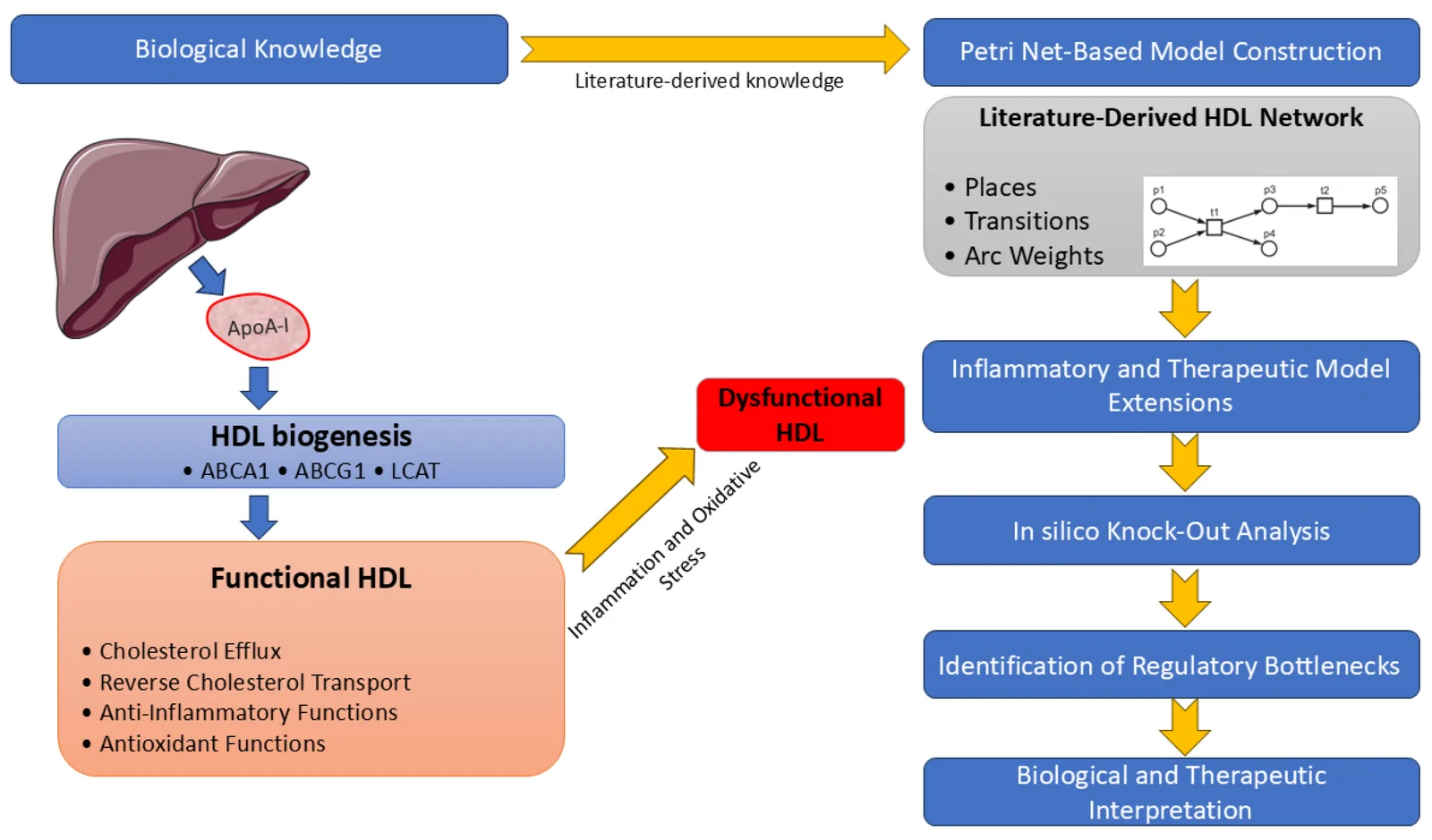
Workflow of the systems-level analysis of HDL dysfunction performed in this study. Current biological knowledge regarding HDL metabolism was used to define the key processes governing ApoA-I-dependent HDL biogenesis and HDL functionality, including cholesterol efflux, reverse cholesterol transport, and anti-inflammatory and antioxidant activities. Exposure of functional HDL to inflammatory and oxidative stress-related mechanisms promotes the development of dysfunctional HDL. This biological knowledge was integrated into a literature-derived HDL network and translated into a Petri net-based model. The model was subsequently extended to represent inflammatory and therapeutic conditions and analyzed using an in silico knock-out approach. This strategy enabled the identification of key regulatory bottlenecks and provided a framework for biological and therapeutic interpretation of HDL dysfunction. Parts of the figure were created using images from Servier Medical Art. Servier Medical Art by Servier is licensed under a Creative Commons Attribution 3.0 Unported License (https://creativecommons.org/licenses/by/3.0/, accessed on 24 August 2026).

To facilitate understanding of both the biological background and the analytical strategy employed in this study, Figure 1 summarizes the overall workflow of the systems-level approach. Current biological knowledge regarding HDL metabolism was used to define the key processes governing ApoA-I-dependent HDL biogenesis and HDL functionality, including cholesterol efflux, reverse cholesterol transport, and anti-inflammatory and antioxidant activities. Since chronic inflammation and oxidative stress promote the transition from functional to dysfunctional HDL, these mechanisms were incorporated into a literature-derived HDL network and subsequently translated into a Petri net-based model. The model was then extended to represent inflammatory and therapeutic conditions and analyzed using an in silico knock-out approach. This workflow enabled the identification of key regulatory bottlenecks governing network organization and provided the basis for the biological and therapeutic interpretation of HDL dysfunction.

Recent evidence suggests that oxidative stress constitutes a critical mechanistic link between lipid metabolism, chronic inflammation, and cardiovascular disease progression, thereby reinforcing the concept of HDL dysfunction as an immunometabolic phenomenon [16]. Inflammatory signaling, oxidative stress, mitochondrial dysfunction, and metabolic reprogramming interact through tightly interconnected regulatory networks that influence both HDL composition and functionality [5,17]. Chronic inflammatory conditions promote the formation of dysfunctional and oxidized HDL particles while simultaneously impairing cholesterol efflux and reverse cholesterol transport [3,5,6,8,18]. Moreover, oxidized HDL has been associated with activation of the NOD-like receptor protein 3 (NLRP3) inflammasome and downstream cytokine signaling, including the release of interleukin-1 beta (IL-1β) and interleukin-18 (IL-18), suggesting that HDL dysfunction may act not only as a consequence, but also as an amplifier of chronic inflammation [19,20].

Collectively, these observations indicate that HDL metabolism cannot be fully understood using a reductionist perspective focused on isolated pathways. Instead, a systems-level approach is required to integrate lipid transport, immune regulation, redox balance, and metabolic adaptation within a common regulatory framework.

We hypothesized that HDL metabolism control is not uniformly distributed throughout the network but is instead concentrated within a limited set of regulatory bottlenecks that determine system behavior under inflammatory and therapeutic conditions.

Here, we present a systematic approach to modeling HDL metabolism using Petri net theory. The aim of the study was to investigate how inflammation and selected therapeutic interventions alter the regulatory organization of HDL metabolism and to identify the processes that exert the greatest control over global network dynamics. To this end, we developed a Petri net-based model representing baseline HDL metabolism and extended it to inflammatory and therapeutic states. Comparative in silico knock-out analysis was then used to determine the functional dependencies between individual processes and the overall behavior of the network, to identify state-specific and conserved regulatory bottlenecks, and to assess how network control is reorganized under pathological and therapeutic conditions. This approach allowed us to examine whether HDL dysfunction can be understood as a systems-level consequence of altered interactions among lipid metabolism, inflammatory signaling, and oxidative stress, and whether therapeutic interventions restore or redirect control within this network.

The remainder of this paper is organized as follows. In Section 2, the proposed model and the outcomes of the analysis are presented. Moreover, the limitations and strengths of the study are discussed in this section. In Section 3, basic notions concerning Petri nets and the construction of Petri net-based models are presented, including the methodology and underlying assumptions. The paper ends with conclusions in Section 4.

## 2. Results and Discussion

### 2.1. Results

This section is divided into two parts. First, the constructed Petri net-based model is presented. Next, the results of the conducted analyses are described.

#### 2.1.1. The Model

**Figure 2:**
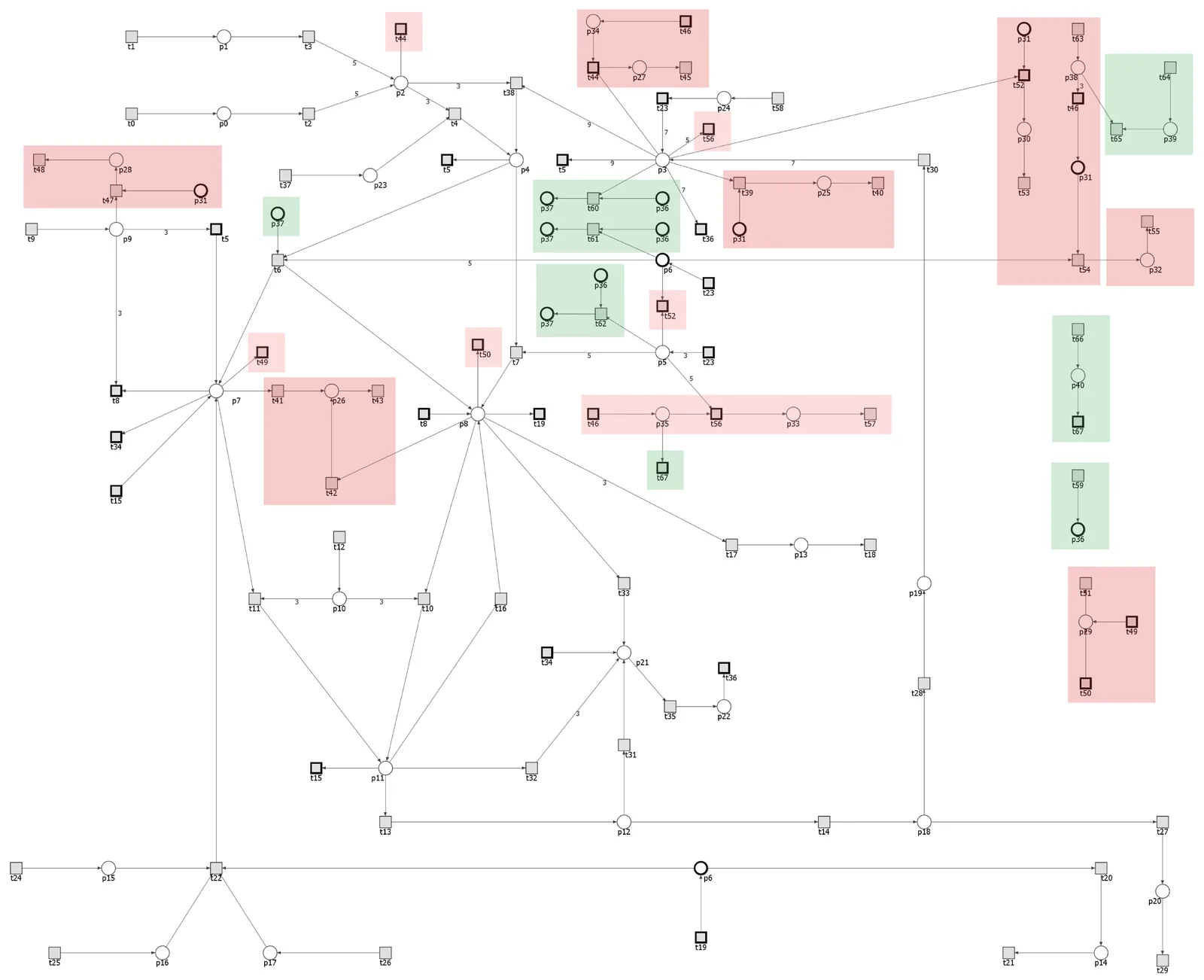
Petri net-based model of HDL metabolism under inflammatory and therapeutic conditions. To improve the readability of the Petri net, logical places and logical transitions (shown in bold) are used as graphical references to existing model elements. They duplicate the graphical representation of the corresponding place or transition, allowing long or intersecting arcs to be omitted. These logical elements are semantically identical to their originals and do not introduce additional places, transitions, or firing behavior. Red highlighted regions denote inflammatory processes, whereas green highlighted regions denote treatment-related processes. The color coding indicates only the process category; it does not represent the direction or magnitude of changes in the corresponding arc weights (i.e., whether weights increase or decrease).

The resulting Petri net-based model, constructed and analyzed using Holmes software (version 2.0) [21], is presented in Figure 2. The model captures the key processes involved in HDL metabolism, as well as their modifications under inflammatory conditions and therapeutic interventions.

A complete list of places representing the system’s elementary components is provided in Table 1. Similarly, the set of transitions, corresponding to elementary interactions and processes within the network, is summarized in Table 2. Together, these elements define the model’s structure and form the basis for subsequent analysis. The net is covered by 17,516 t-invariants and 19 Minimal Common Transition (MCT) sets. However, the MCT sets provide limited insight: 17 consist of only two transitions, while the remaining sets contain four and three transitions, respectively. The remaining 27 transitions do not belong to any MCT set. The model is available in the Supplementary Materials.

#### 2.1.2. Results of In Silico Knock-Out Analysis

The results of the in silico knock-out analysis are summarized by comparing system behavior after disabling individual transitions with the corresponding reference models. Changes in token distribution and flow were used as a measure of each transition’s influence on global network dynamics. This analysis was performed separately for the inflammatory model and the inflammation-with-treatment model to assess context-dependent control mechanisms.

In the inflammatory model, knock-out analysis revealed that system behavior is predominantly controlled by transitions associated with impaired HDL maturation and inflammation-driven dysfunction. Disabling transitions related to reduced LCAT-mediated cholesterol esterification ($t_{47}$), adiponectin decreased synthesis ($t_{44}$), and oxidized HDL-associated activity decrease ($t_{56}$) resulted in pronounced redistribution of token flow across multiple pathways, including HDL remodeling, cholesterol efflux, and inflammatory signaling. These transitions exerted strong system-wide effects, indicating their central role in maintaining the network’s pathological inflammatory state.

Following the introduction of therapeutic mechanisms, the sensitivity of the system to transition knock-outs changed markedly. In the treatment model, the most influential transitions were those directly associated with therapeutic effect, including inhibition of inflammation ($t_{65}$), reduction of oxidative stress and restoration of redox balance ($t_{67}$), and reconstituted HDL-mediated cholesterol export via ABCA1, SR-B1, and ABCG1 ($t_{60}$–$t_{62}$). Knock-out of these transitions led to substantial destabilization of the network, as reflected by a loss of cholesterol efflux efficiency and the re-emergence of inflammation-driven token accumulation.

Comparison of the two model variants demonstrated that the sets of key regulatory transitions differ substantially between the inflammatory and therapeutic contexts. These findings indicate a context-dependent reorganization of control within the HDL metabolic network, reflecting a shift from pathology-driven regulation under inflammatory conditions to therapy-dependent stabilization mechanisms after intervention.

The most influential transitions identified for each model variant are summarized in Table 3.

Unlike the inflammatory model, which is dominated by HDL dysfunction, impaired maturation, and oxidative stress-related mechanisms, the therapeutic model integrates three complementary restoration pathways: inhibition of inflammation, reduction of oxidative stress with restoration of redox balance, and enhancement of cholesterol efflux through rHDL-mediated transport.

Key transition sets differ between the inflammatory and therapeutic models, reflecting a shift from inflammation-driven destabilization of HDL metabolism to therapy-dependent restoration of cholesterol efflux and redox balance.

**Figure 3:**
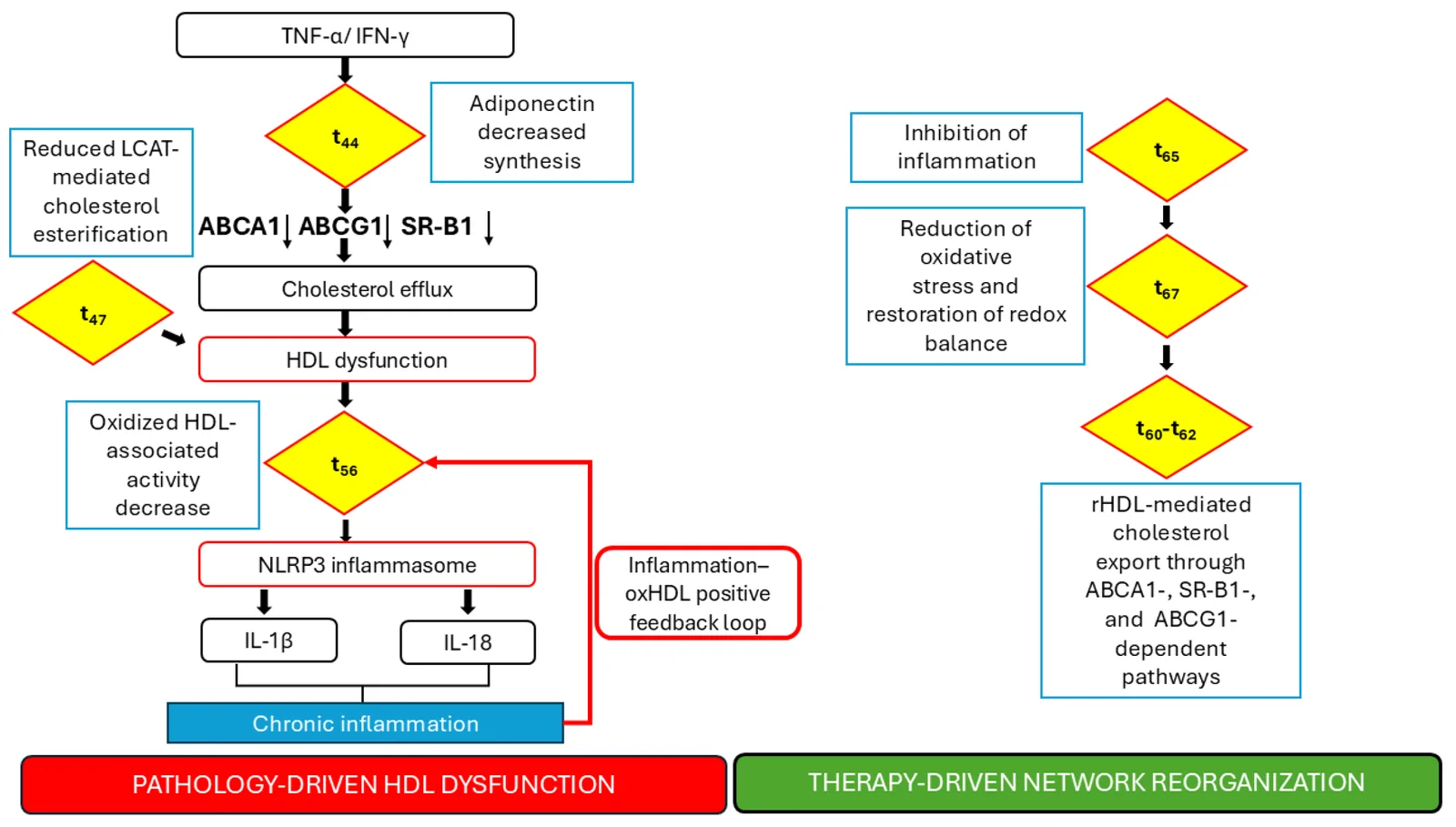
Regulatory bottlenecks governing pathology-driven and therapy-driven organization of the HDL metabolic network. Highlighted bottlenecks correspond to the key regulatory transitions identified by the in silico knock-out analysis: $t_{44}$ (adiponectin decreased synthesis), $t_{47}$ (reduced LCAT-mediated cholesterol esterification), $t_{56}$ (oxidized HDL-associated activity decrease), $t_{65}$ (inhibition of inflammation), $t_{67}$ (reduction of oxidative stress and restoration of redox balance), and $t_{60}$–$t_{62}$ (rHDL-mediated cholesterol export through ABCA1-, SR-B1-, and ABCG1-dependent pathways). Under inflammatory conditions, TNF-α and IFN-γ promote adiponectin-dependent dysregulation ($t_{44}$), leading to reduced ABCA1-, ABCG1-, and SR-B1-mediated cholesterol efflux and progressive HDL dysfunction. Reduced LCAT-mediated cholesterol esterification ($t_{47}$) further impairs HDL maturation, whereas oxidized HDL-associated activity decrease ($t_{56}$) contributes to NLRP3 inflammasome activation, IL-1β and IL-18 signaling, and the establishment of a positive feedback loop linking oxidative stress, inflammation, and HDL dysfunction. Together, these mechanisms stabilize a pathology-driven network state characterized by impaired cholesterol transport and chronic inflammatory activation. Following therapeutic intervention, network control is reorganized toward inhibiting inflammation ($t_{65}$) and reducing oxidative stress, with restoration of redox balance ($t_{67}$). In parallel, rHDL-mediated cholesterol export pathways ($t_{60}$–$t_{62}$) restore ABCA1-, SR-B1-, and ABCG1-dependent cholesterol efflux. Collectively, these processes promote recovery of HDL functionality and stabilization of the metabolic network. The identified bottlenecks represent the principal regulatory processes emerging from the model and illustrate the transition from pathology-driven dysfunction to therapy-driven restoration of network homeostasis.

To facilitate biological interpretation of the identified control structure, the principal regulatory bottlenecks and their role in pathology-driven and therapy-driven network organization are summarized schematically in Figure 3.

#### 2.1.3. System-Wide Effects Revealed by Heatmap Analysis

**Figure 4:**
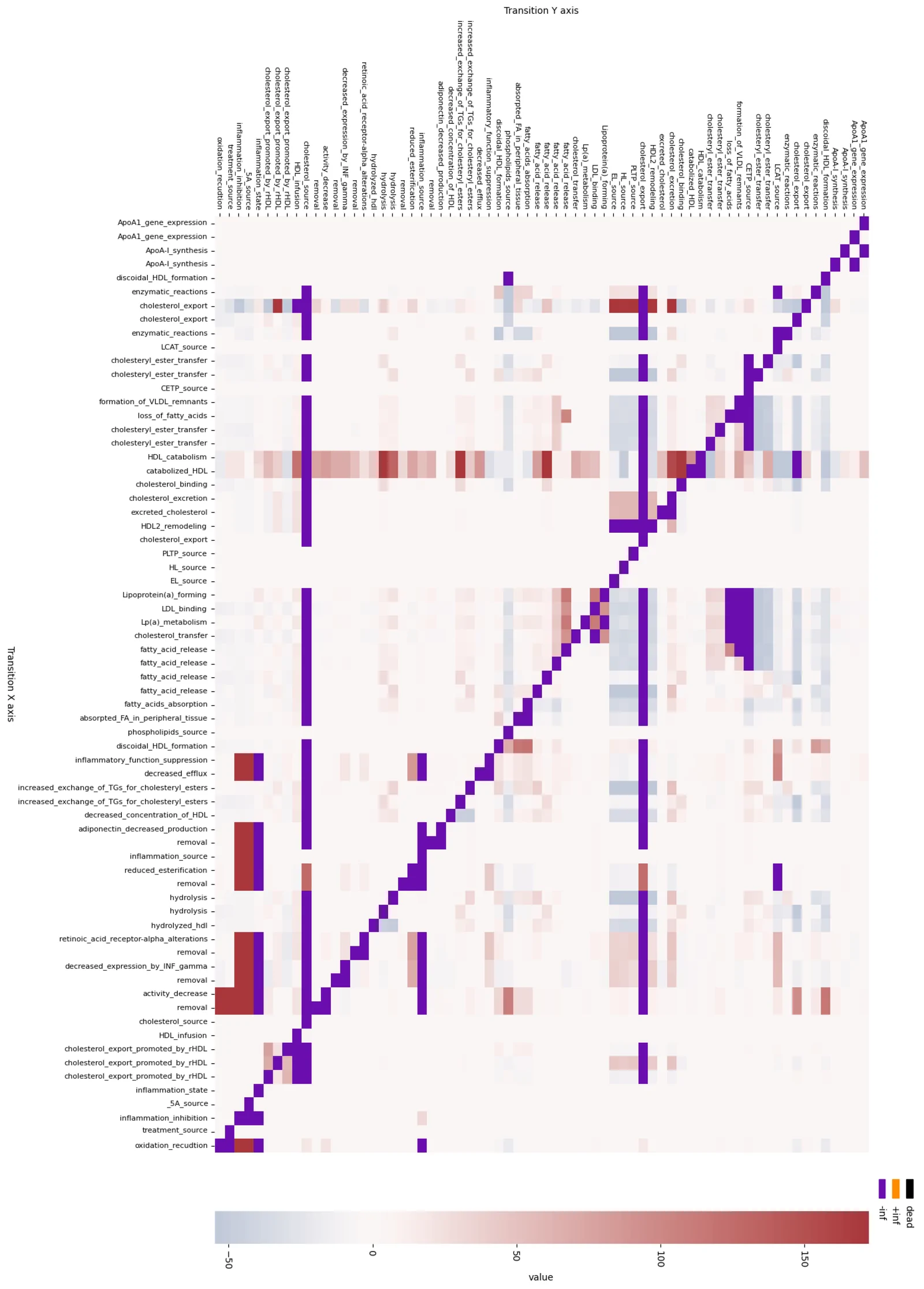
Heatmap of in silico knock-out analysis results. Values represent the mean percentage difference in token flow through each transition between the knock-out and reference models, averaged across repeated runs; no additional normalization was applied. Positive values (red shades) indicate increased token flow relative to the reference model, whereas negative values (blue shades) indicate reduced token flow. Stronger effects correspond to larger absolute percentage differences, reflected by greater color intensity. Values marked as *+inf* correspond to transitions that become active only in the knock-out scenario, while *-inf* indicates complete loss of activity when a given transition is disabled. Transitions marked as *dead* would correspond to transitions inactive in both reference and knock-out conditions; such cases do not occur in the present model.

To further characterize the global impact of individual transition knock-outs on network behavior, the results were visualized using a heatmap representation (Figure 4).

The heatmap compares token flow in each knock-out scenario with the corresponding reference model, providing an overview of how disabling individual transitions affects the system at a network-wide level.

The majority of transition knock-outs resulted in modest and localized changes, indicating a high degree of robustness within large parts of the network. In contrast, a limited subset of knock-outs produced pronounced and widespread redistribution of token flow, affecting multiple pathways related to HDL remodeling, cholesterol transport, and inflammatory signaling. These cases are reflected by strong positive or negative deviations in the heatmap, highlighting transitions with system-wide regulatory influence.

Notably, transitions associated with cholesterol esterification (LCAT-related processes), ABCA1- and ABCG1-mediated cholesterol efflux, and rHDL-dependent export exhibited particularly strong effects, consistent with their central role in controlling HDL metabolism. The heatmap thus provides a global context for identifying key regulatory transitions summarized in Section 2.1.2 and supports the conclusion that control over the network is highly uneven among its components.

### 2.2. Discussion

As illustrated in Figure 3, inflammatory conditions reorganize network control around a limited set of bottleneck transitions associated with adiponectin decreased synthesis ($t_{44}$), reduced LCAT-mediated cholesterol esterification ($t_{47}$), and oxidized HDL-associated activity decrease ($t_{56}$), whereas therapeutic interventions shift control towards inhibition of inflammation ($t_{65}$) and reduction of oxidative stress and restoration of redox balance ($t_{67}$).

A central insight emerging from this study is that control over HDL metabolism is highly unevenly distributed and organized around a small number of key regulatory transitions. As shown in Table 3, only a limited subset of processes exerts a dominant influence on global network behavior, supporting the concept of regulatory bottlenecks in HDL metabolism.

In the inflammatory state, these bottlenecks are reduced LCAT-mediated cholesterol esterification ($t_{47}$), oxidized HDL-associated activity decrease ($t_{56}$), and adiponectin decreased synthesis ($t_{44}$). Together, these processes define a pathology-driven control regime in which HDL functionality is destabilized and shifts toward a pro-inflammatory phenotype.

By contrast, in the therapeutic context, the control structure undergoes a pronounced reorganization toward processes that suppress inflammation and restore redox balance. Transitions such as inflammation inhibition ($t_{65}$) and reduction of oxidative stress and restoration of redox balance ($t_{67}$) emerge as dominant regulatory nodes, indicating that effective interventions exert system-wide effects by reconfiguring the network’s global structure rather than acting on isolated pathways.

The recurring importance of inflammation and redox regulation across both model contexts suggests that these processes constitute a shared regulatory framework governing HDL network organization despite large-scale reorganization of peripheral processes. This observation has important conceptual implications, indicating that therapeutic strategies targeting these processes may achieve system-wide effects disproportionate to their local action.

Taken together and summarized in Table 4, these findings demonstrate that HDL metabolism should be understood not as a collection of parallel pathways, but as a hierarchically organized network, in which a small number of processes control overall system behavior.

The present study provides a systems-level perspective on HDL metabolism under inflammatory and therapeutic conditions, demonstrating that control over HDL functionality emerges from the dynamic organization of interacting processes rather than from isolated molecular components. By integrating established biological knowledge into a Petri net framework and applying in silico knock-out analysis, we identified context-dependent regulatory patterns that are difficult to capture using non-systems approaches.

A central finding of this work is that the set of transitions exerting the strongest control over network behavior differs markedly between the inflammatory and treatment models. In the inflammatory state, system dynamics are primarily governed by processes associated with impaired HDL maturation and inflammation-driven dysfunction, including reduced LCAT-mediated cholesterol esterification, decreased adiponectin-dependent regulation, and loss of oxidized HDL-related activity. These observations are consistent with experimental and clinical evidence demonstrating that chronic inflammation induces structural remodeling and functional impairment of HDL particles, promoting a shift from an atheroprotective to a pro-inflammatory phenotype [5,6,7,8].

The central role of oxidized HDL identified in the present model is consistent with emerging evidence linking HDL dysfunction to NLRP3 inflammasome activation. Oxidized HDL particles have been shown to promote inflammasome signaling, resulting in the release of IL-1β and IL-18 and further impairment of cholesterol efflux pathways mediated by ABCA1 and ABCG1 [19,20,22]. In the present study, oxidized HDL (oxHDL) is interpreted as a functional HDL state resulting from oxidative modification of HDL components, including ApoA-I and HDL-associated lipids. Such modifications impair cholesterol efflux pathways, alter HDL remodeling, and promote pro-inflammatory signaling. Consequently, oxHDL represents a dysfunctional HDL phenotype that may contribute to the amplification of vascular and inflammatory responses. From a systems perspective, this creates a self-reinforcing feedback loop in which oxidative stress, inflammation, and HDL dysfunction mutually amplify one another. The identification of oxidation-related transitions among the key regulatory bottlenecks therefore supports the view that inflammasome-associated signaling may represent an important mechanistic link between lipid dysregulation and chronic inflammatory states.

Since IL-18 is a mediator connecting oxidative stress, vascular inflammation, and atherosclerotic progression, its involvement suggests that HDL dysfunction may contribute to broader immunometabolic regulatory networks [23,24].

Emerging concepts of trained immunity further support the view that lipid metabolism, inflammatory signaling, and cellular metabolic reprogramming form an integrated regulatory network rather than independent biological processes [25]. Within this framework, persistent inflammatory stimuli may induce long-lasting functional changes in innate immune cells, contributing to chronic low-grade inflammation and sustained alterations in HDL functionality. These observations further support the interpretation of HDL metabolism as an adaptive immunometabolic system rather than a collection of isolated biochemical pathways. Recent lipidomic studies support this perspective, demonstrating that HDL particles undergo extensive compositional remodeling under metabolic stress and inflammatory conditions. These alterations extend far beyond changes in HDL-C concentration and affect multiple lipid species involved in cholesterol transport, redox balance, and immune regulation, reinforcing the concept that HDL should be viewed as a complex functional system rather than a simple cholesterol carrier [17,26].

Following the introduction of therapeutic mechanisms, the control structure of the network undergoes a pronounced reorganization. In the treatment model, dominance shifts toward transitions directly associated with therapeutic modulation, including inhibition of inflammation, reduction of oxidative stress, and rHDL-mediated cholesterol export via ABCA1-, SR-B1-, and ABCG1-dependent pathways [27,28]. This result supports the concept that effective HDL-targeted therapies do not simply restore individual pathways but rather reconfigure the global balance between competing metabolic and inflammatory processes. Such network-level reorganization aligns with growing evidence that therapeutic benefit depends on improving HDL functionality rather than merely increasing HDL concentration [9,10,11,12,29,30]. Recent analyses of the HDL lipidome further indicate that profound compositional remodeling of HDL particles occurs in metabolic disorders, emphasizing that functional and structural HDL characteristics may be more informative than HDL-C levels alone [26]. This interpretation is further supported by clinical experience with CETP inhibitors, which has shown that substantial increases in HDL-C concentration do not necessarily translate into proportional cardiovascular benefit, underscoring the importance of HDL quality and biological activity over HDL-C levels alone [12,29].

The limited overlap between key regulatory transitions across inflammatory and therapeutic contexts, restricted mainly to inflammation inhibition and oxidation reduction, highlights the adaptive nature of HDL metabolism. While certain core processes remain universally important, most control mechanisms appear to be highly context dependent. Biologically, this underscores the dual role of inflammation: both a disruptor of HDL homeostasis and a target that, when modulated, can restore network stability. From a modeling standpoint, it illustrates how Petri net-based approaches capture shifts in system control that are not evident at the level of individual reactions.

The heatmap analysis further reinforces this interpretation by revealing a strongly uneven distribution of control across the network. Many knock-outs resulted in localized or modest effects, while a small subset of transitions caused a profound, system-wide redistribution of token flow, simultaneously affecting HDL remodeling, cholesterol transport, and inflammatory signaling. Such disproportionate influence of selected processes is a hallmark of complex biological networks and has been reported in other Petri net-based analyses of metabolic and regulatory systems [21,31]. These findings validate knock-out-driven sensitivity analysis as an effective strategy to identify regulatory bottlenecks in large-scale biological networks.

More broadly, this study supports the view that Petri nets constitute a particularly suitable formalism for modeling immunometabolic systems characterized by concurrency, feedback loops, and non-linear interactions. Previous Petri net-based models developed by Formanowicz et al. have demonstrated context-dependent control structures in whole-body homeostatic and inflammation-driven processes, including iron homeostasis, anemia of chronic disease, and essential hypertension [32,33,34,35]. Our results extend this line of research to HDL metabolism, highlighting how Petri nets can bridge molecular mechanisms, systems behavior, and therapeutic intervention within a unified computational framework.

From a conceptual perspective, the present findings are consistent with recent systems-oriented views that identify oxidative stress and inflammation as central hubs driving chronic disease states [17,22,23,36]. Modeling HDL metabolism within this framework reveals disease and treatment states as distinct organizational regimes of the same underlying network, explaining why interventions effective in one context may fail in another and underscoring the importance of context-aware therapeutic design.

This systems-level interpretation may be particularly relevant for chronic immunometabolic disorders characterized by persistent oxidative stress and inflammation, such as metabolic syndrome, diabetes mellitus, and chronic kidney disease. In these conditions, extensive HDL remodeling and impaired cholesterol efflux have been described, suggesting that the regulatory bottlenecks identified here may represent shared mechanisms governing network dysfunction across multiple chronic disease states.

### 2.3. Limitations and Strengths of the Study

Several limitations of this study should be acknowledged. The model is qualitative and relies on literature-based assumptions regarding the direction and relative strength of interactions rather than on quantitative kinetic parameters. While this approach is well-suited for identifying control structures and system-level dependencies, it does not aim to predict absolute concentrations or temporal dynamics. Future work incorporating quantitative data or stochastic Petri nets could enhance predictive capability. Moreover, the therapeutic mechanisms included here represent selected strategies rather than an exhaustive catalog of possible interventions.

Moreover, the proposed Petri net model represents a qualitative, literature-derived abstraction of HDL metabolism and its interactions with inflammatory and oxidative stress pathways. As such, it is intended to identify regulatory dependencies and system-level control structures rather than predict absolute concentrations, kinetic parameters, or temporal dynamics.

Furthermore, the model incorporates selected biological processes and therapeutic mechanisms considered particularly relevant to HDL dysfunction, whereas additional molecular interactions may also contribute to network behavior.

Some regulatory relationships included in the model are supported predominantly by experimental evidence and may require further validation in human studies. The present model focuses on the principal processes governing HDL functionality and therefore does not explicitly incorporate all HDL-associated proteins, including ApoA-II, ApoC family proteins, serum amyloid A (SAA), and other components of the HDL proteome. These molecules may influence HDL remodeling, acute-phase HDL formation, cholesterol efflux, and overall HDL functionality and therefore represent potential extensions of future model versions. Their exclusion from the current model should not be interpreted as evidence of limited biological importance but rather as a consequence of the level of abstraction required for the present systems-level analysis.

The model also represents oxidized HDL (oxHDL) as a functional state characterized by impaired cholesterol efflux, reduced antioxidant capacity, and enhanced pro-inflammatory activity rather than explicitly distinguishing between oxidation of HDL lipids and proteins. Incorporation of these molecular details may further refine future model versions. In addition, the current model focuses primarily on HDL-centered processes and does not explicitly represent LDL retention, LDL oxidation, or their potential effects on the regulation of cholesterol transporters and HDL biogenesis.

Several biological mechanisms incorporated into the model are derived from experimental and animal studies. Because HDL composition, apolipoprotein distribution, and HDL functionality differ substantially among species, particularly between humans and commonly used experimental models, the translation of these findings to human biology should be interpreted with appropriate caution.

Despite these limitations, the study has several important strengths. By integrating HDL metabolism, inflammation, and oxidative stress into a unified systems-level framework, the model enables identification of regulatory bottlenecks that are difficult to detect with reductionist approaches focused on isolated pathways. The Petri net formalism provides a transparent representation of network organization and supports systematic in silico perturbation analyses, allowing evaluation of the relative importance of individual processes within the network. Furthermore, the identified regulatory bottlenecks provide biologically plausible hypotheses that may guide future experimental investigations and contribute to the development of function-oriented therapeutic strategies targeting HDL dysfunction.

Overall, the present study supports the view that HDL dysfunction arises from complex interactions among lipid metabolism, inflammatory signaling, and oxidative stress, highlighting the value of systems-level approaches for investigating immunometabolic disorders.

## 3. Materials and Methods

### 3.1. Basics of Petri Nets

Petri nets are mathematical objects that can be used to model and analyze concurrent processes. They have been developed in the context of modeling technical systems, especially those related to information processing. However, such nets are increasingly used to model and analyze complex biological systems.

A Petri net has a structure of a directed bipartite graph, i.e., it is composed of two disjoint sets of vertices and arcs connecting them. Vertices belonging to one of these sets are called places, while those being elements of the other set are transitions. The arcs can connect only places to transitions, or transitions to places. Places correspond to passive components of the modeled system, such as chemical compounds, whereas transitions correspond to active components, such as chemical reactions. The arcs represent causal relations between the passive and active components. Positive integer weights are associated with the arcs [31,37].

In addition to vertices and arcs, there are also tokens in Petri nets. They are located in places and represent amounts of the respective passive components. Tokens can flow from one place to another through transitions, and this flow corresponds to the flow of signals in the modeled system [31,37].

Due to tokens, Petri nets can be used not only to model the structure of the system but also to model the dynamics occurring in the system.

The flow of tokens follows a simple rule called the transition firing rule. According to this rule, a transition is active if in each of the places directly preceding it (i.e., a place from which an arc leading to this transition originates) the number of tokens is at least equal to the weight of the arc connecting that place to the transition. An active transition can fire, which means that tokens can flow from the places directly preceding it to those directly following it. The number of flowing tokens is equal to the weights of respective arcs [31,37].

Petri nets have a very intuitive graphical representation, which helps to understand the structure of the modeled system. In this representation, places are depicted as circles, transitions as rectangles, and tokens as dots or numbers located in places. Although this representation is intuitive, it is not well-suited to analyzing the formal properties of the nets. For this purpose, an incidence matrix is used. In such a matrix $A_{n\times m}$, rows correspond to places while columns correspond to transitions, where *n* and *m* are the numbers of places and transitions, respectively, in the Petri net. A number located in the *i*-th row and *j*-th column of the matrix is equal to the difference between the numbers of tokens in place $p_{i}$ after and before the firing of transition $t_{j}$.

Based on the incidence matrix, t-invariants can be calculated. They are vectors of natural numbers $x\in N^{m}$ satisfying the matrix equation A·x=0. While transitions correspond to elementary processes in the modeled system, t-invariants correspond to subprocesses which do not change the state of the system.

To each t-invariant *x* there corresponds its support $s\left(x\right)=\left\{t_{j}:x_{j}>0,j=0,1,2,\ldots ,m\right\}$. A Petri net, being a model of a biological system, should be covered by t-invariants, meaning each transition should belong to a support of at least one t-invariant [31,37].

Transitions can be grouped into sets called MCT sets. Such a set contains those transitions that belong to the supports of exactly the same t-invariants. MCT sets divide the set of transitions into disjoint subsets. Each of them corresponds to a functional block of the modeled system (except the single-element MCT sets) [38].

An analysis of a Petri net-based model of a biological system can be based on t-invariants or other formal properties of the net. It can also be based on simulation. In this case, a knockout analysis is especially interesting. It is possible to switch off selected transitions and observe the effect of this on the rest of the net. In this case, the role of selected elementary processes in the modeled system can be investigated [39].

### 3.2. Construction of Petri Net-Based Models

**Figure 5:**
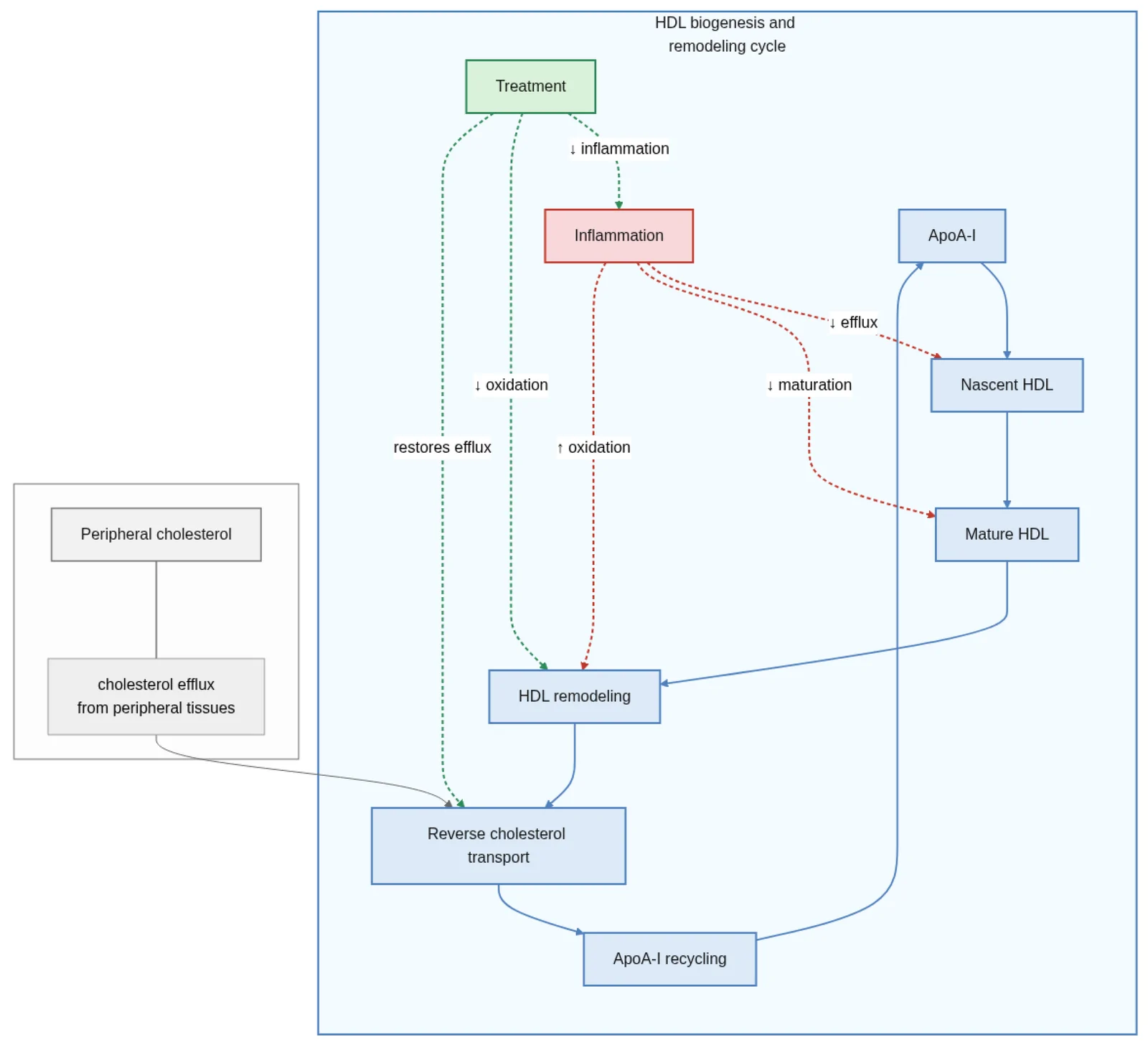
Conceptual overview of the HDL biogenesis and remodeling cycle and its modulation by inflammation and therapeutic interventions. Under physiological conditions, ApoA-I undergoes HDL formation, maturation, remodeling, reverse cholesterol transport, and recycling. Inflammation impairs HDL maturation and cholesterol efflux while promoting HDL oxidation, resulting in dysfunctional HDL particles. Therapeutic interventions counteract these alterations by reducing inflammation and oxidation and by restoring cholesterol efflux. The figure provides a simplified representation of the biological processes included in the Petri net model.

The Petri net-based model was developed in a stepwise manner to progressively capture increasing levels of biological complexity associated with HDL metabolism. The construction process consisted of three main stages: (i) modeling of baseline HDL metabolism, (ii) incorporation of inflammatory processes, and (iii) implementation of therapeutic interventions. An overview of the modeled HDL biogenesis and remodeling processes together with the effects of inflammation and therapeutic interventions is presented in Figure 5. The diagram summarizes the principal biological mechanisms incorporated into the Petri net model and illustrates their interactions at a conceptual level.

In the first stage, a core model of HDL metabolism was established based on selected literature describing key pathways and mechanisms involved in HDL formation, remodeling, and clearance [5,10,40]. The aim of this step was to capture the essential processes governing HDL dynamics under physiological conditions. The resulting network included major components such as lipid transport, apolipoprotein synthesis, enzymatic modification, lipoprotein remodeling, and cholesterol clearance.

HDL biogenesis is initiated by the synthesis and release of ApoA-I from the liver ($p_{0}$) and intestine ($p_{1}$), represented by transitions corresponding to ApoA-I synthesis and secretion ($t_{2}$, $t_{3}$). A small fraction of circulating ApoA-I exists in a lipid-poor form ($p_{2}$) and serves as an acceptor for cholesterol and phospholipids exported from peripheral cells during HDL biogenesis.

The initial lipidation step is mediated primarily by ABCA1 ($p_{3}$), which facilitates cholesterol and phospholipid efflux toward ApoA-I through transitions representing nascent HDL formation ($t_{4}$, $t_{38}$). These interactions lead to the formation of discoidal HDL particles ($p_{4}$), which represent structurally immature but functionally active HDL forms that play a key role in cellular cholesterol acceptance and the early stages of reverse cholesterol transport.

Subsequent maturation of HDL particles is driven by LCAT ($p_{9}$). LCAT-mediated esterification of free cholesterol is represented by enzymatic transitions ($t_{5}$, $t_{8}$), enabling the transition from discoidal HDL ($p_{4}$) to spherical HDL particles ($p_{7}$, $p_{8}$). As HDL circulates, additional cholesterol is exported from peripheral tissues via ABCA1 ($p_{3}$) and ABCG1 ($p_{5}$), further contributing to HDL growth and remodeling.

HDL remodeling is further governed by lipid exchange processes. CETP ($p_{10}$) mediates the exchange of cholesteryl esters and triglycerides between HDL and apoB-containing lipoproteins through transitions $t_{10}$ and $t_{11}$. PLTP ($p_{15}$) contributes to phospholipid redistribution, while hepatic lipase ($p_{16}$) and endothelial lipase ($p_{17}$) are represented by transitions associated with HDL hydrolysis and remodeling ($t_{49}$, $t_{50}$). These processes jointly determine HDL size distribution and functional heterogeneity.

Selective cholesterol uptake from HDL is mediated by SR-B1 ($p_{6}$) via cholesterol export transitions ($t_{6}$, $t_{23}$). The HDL life cycle is completed by catabolic processes, including HDL degradation and lipid-poor ApoA-I clearance through the kidneys ($p_{14}$), represented by cholesterol excretion and removal transitions ($t_{20}$–$t_{22}$).

In the second stage, inflammatory processes were incorporated into the model to represent the impact of inflammation on HDL metabolism. Inflammation was introduced by adding dedicated places and transitions, as well as by modifying the arc weights of existing processes, thereby reflecting experimentally observed inflammation-induced alterations. These modifications include reduced transporter activity (ABCA1, ABCG1, SR-B1), impaired LCAT-mediated cholesterol esterification, increased HDL oxidation, enhanced hydrolysis, and altered lipid exchange dynamics. Key inflammation-related transitions include adiponectin decreased synthesis ($t_{44}$), reduced LCAT-mediated cholesterol esterification ($t_{47}$), oxidized HDL-associated activity decrease ($t_{56}$), inflammatory function suppression ($t_{39}$), and cytokine-driven expression changes ($t_{52}$, $t_{54}$). A detailed summary of all inflammation-related modifications and their literature justification is provided in Table 5.

In the final stage, therapeutic interventions were incorporated into the model by introducing additional transitions or modifying the weights of existing arcs. These interventions include reconstituted HDL-mediated cholesterol export ($t_{60}$–$t_{62}$), inhibition of inflammation ($t_{65}$), reduction of oxidative stress and restoration of redox balance (e.g., vitamins C and E effects, $t_{67}$), and peptide-based anti-inflammatory therapy ($p_{39}$).

The complete set of therapeutic modifications and their mechanistic justification is summarized in Table 6.

This subsection focuses on the formal and structural aspects of the Petri net construction. A detailed biological interpretation of the resulting HDL inflammation and treatment model is provided in the following subsection.

### 3.3. Biological Rationale of the HDL Inflammation and Treatment Model

The Petri net model developed in this study represents HDL metabolism as a dynamic, context-dependent biological network modulated by inflammation and therapeutic interventions. Rather than treating HDL as a homogeneous lipoprotein fraction, the model captures the continuous structural and functional remodeling of HDL particles and integrates metabolic, inflammatory, and regulatory processes acting across multiple tissues.

The baseline structure of the model reflects the canonical pathways of HDL biogenesis, maturation, and clearance. HDL formation is initiated by the synthesis and release of ApoA-I from the liver and intestine. A small fraction of circulating ApoA-I exists in a lipid-poor form and participates in HDL biogenesis. In the circulation, ApoA-I serves as an acceptor of cholesterol and phospholipids exported from peripheral cells, primarily via ABCA1- and ABCG1-dependent mechanisms. This lipidation process leads to the formation of nascent, discoidal HDL particles. Subsequent maturation of HDL is driven by LCAT, which esterifies free cholesterol, enabling the transition from discoidal to spherical HDL particles. The model further incorporates continuous HDL remodeling mediated by cholesteryl ester transfer protein, phospholipid transfer protein, and lipases, as well as hepatic uptake and renal processing of HDL components, thereby capturing the full life cycle of HDL particles.

Inflammatory conditions are introduced into the model as system-level perturbations that alter multiple processes simultaneously. Inflammation affects HDL metabolism by impairing cholesterol efflux efficiency, reducing transporter expression and activity, suppressing LCAT-mediated esterification, and promoting the formation of oxidized and structurally dysfunctional HDL. Pro-inflammatory cytokines, including TNF-α and IFN-γ, are known to modulate these processes by decreasing adiponectin production, altering nuclear receptor signaling, and reducing the expression of HDL-related transporters. As a result, HDL particles shift functionally from atheroprotective, anti-inflammatory forms to forms with diminished cholesterol-transport capacity and increased pro-inflammatory potential. Feedback mechanisms are also represented, whereby dysfunctional HDL further sustains inflammatory signaling.

The therapeutic extension of the model incorporates interventions targeting both inflammatory signaling and HDL functionality. rHDL is represented as an exogenous acceptor of cholesterol that enhances cholesterol export via ABCA1-, ABCG1-, and SR-B1-dependent pathways. The peptide 5A is modeled as an inhibitor of inflammatory and oxidative processes, attenuating the dominance of pro-inflammatory transitions within the network. The antioxidant effects of vitamins C and E are introduced as mechanisms that reduce HDL oxidation and restore redox balance. Collectively, these interventions shift the system from inflammation-driven dysregulation toward a state in which cholesterol efflux and lipid homeostasis are partially restored.

From a systems perspective, the model describes HDL metabolism as an adaptive network in which the relative influence of individual processes depends on the biological context. Under inflammatory conditions, network behavior is dominated by transitions associated with impaired esterification, oxidative stress, and inflammatory signaling. Following the introduction of therapeutic mechanisms, the network’s control structure reorganizes toward processes that inhibit inflammation, reduce oxidation, and promote cholesterol export. This context-dependent organization provides the biological foundation for subsequent in silico knock-out analyses.

### 3.4. In Silico Knock-Out Analysis

To identify processes exerting the greatest control over system behavior, an in silico knock-out analysis was performed. In this approach, individual transitions within the Petri net were systematically disabled to simulate the functional removal or inhibition of specific biological processes. For each knock-out scenario, the resulting system behavior was compared with that of the corresponding reference model.

For each transition knock-out, simulations were performed using the knock-out simulation functionality implemented in Holmes. Each simulation consisted of 1000 simulation steps and was repeated 100 times to account for the stochastic nature of the model. The knock-out and corresponding reference simulations were initialized using the same starting conditions, with the only difference being the disabling of the transition under investigation. Results obtained across the 100 repetitions were averaged, and the effect of each knock-out was evaluated by comparing the mean activities of the remaining transitions with their mean activities in the corresponding reference simulations.

The impact of each transition knock-out was quantified by analyzing changes in token distribution and flow throughout the network. Differences between knock-out and reference simulations were expressed as mean percentage changes in transition activity. A knock-out was considered to have a substantial effect when it produced a pronounced redistribution of activity across multiple transitions in the network, rather than on the basis of a large change in a single transition alone. Thus, both the magnitude of the observed changes and the number of affected transitions were taken into account. In some cases, individual transition activities changed several-fold, including changes exceeding tenfold relative to the reference model, whereas in other cases more moderate changes were considered substantial when they occurred consistently across a larger group of transitions. This analysis was performed separately for the inflammatory model and the inflammation-with-treatment model, allowing direct comparison of control structures under disease and therapeutic conditions.

Transitions whose knock-out resulted in substantial redistribution of token flow were classified as key regulatory elements of the system. This strategy enabled the identification of context-dependent control mechanisms governing HDL metabolism and provided the basis for interpreting how inflammation and therapeutic interventions reorganize network regulation.

In the final stage, selected therapeutic strategies targeting HDL metabolism and inflammation were incorporated into the model. These interventions were modeled as additional transitions or modifications of existing processes, enabling simulation of their potential effects on the system. The goal of this step was to assess how different therapeutic approaches can influence HDL functionality and inflammatory responses. A comprehensive list of therapies implemented and their mechanistic justification is presented in Table 6.

## 4. Conclusions

This work shows that HDL metabolism should not be interpreted as a collection of isolated biochemical pathways but rather as a dynamic, context-dependent immunometabolic network governed by interacting regulatory processes. Using a Petri net-based systems approach, we demonstrate that inflammatory conditions reorganize the control structure of HDL metabolism, shifting dominance toward processes associated with oxidative stress, reduced LCAT-mediated cholesterol esterification, and disrupted metabolic signaling. In contrast, therapeutic interventions act by reconfiguring network-level regulation, promoting cholesterol efflux, and reducing inflammatory and oxidative burden.

Importantly, only a limited subset of processes, particularly inflammation and oxidation, remained central across both pathological and therapeutic states. Transitions representing these processes and the related therapeutic mechanisms constitute a core regulatory backbone of the system and represent potential bottlenecks governing HDL functionality.

Overall, our findings support a systems-oriented view of HDL biology in which functionality emerges from the interplay between lipid metabolism, immune signaling, and redox balance. This perspective may help explain the limited success of therapies focused exclusively on increasing HDL levels and suggests that effective interventions should target network-level regulation rather than individual molecular components.

## Figures and Tables

**Table 1:** List of places.

Place	Biological Meaning
$p_{0}$	liver
$p_{1}$	intestine
$p_{2}$	lipid-poor ApoA-I involved in HDL biogenesis
$p_{3}$	ABCA1
$p_{4}$	discoidal HDL
$p_{5}$	ABCG1
$p_{6}$	SR-B1
$p_{7}$	spherical HDL3
$p_{8}$	spherical HDL2
$p_{9}$	LCAT
$p_{10}$	CETP
$p_{11}$	VLDL
$p_{12}$	Intermediate-Density Lipoprotein (IDL)
$p_{13}$	High-Density Lipoprotein Receptor (HDLR)
$p_{14}$	kidney
$p_{15}$	PLTP
$p_{16}$	Hepatic Lipase (HL)
$p_{17}$	Endothelial Lipase (EL)
$p_{18}$	LDL
$p_{19}$	Low-Density Lipoprotein Receptor (LDLR)
$p_{20}$	Lipoprotein(a) (Lp(a))
$p_{21}$	fatty acid (FA)
$p_{22}$	Cluster of Differentiation 36 (CD36)
$p_{23}$	phospholipid
$p_{24}$	cholesterol
$p_{25}$	impaired cholesterol
$p_{26}$	Triglyceride (TG)-enriched HDL-C clearance
$p_{27}$	decreased expression
$p_{28}$	reduced formation of HDL-C
$p_{29}$	HDL hydrolysis increased by secretory phospholipase A2 (sPLA2)
$p_{30}$	decreased expression
$p_{31}$	inflammation
$p_{32}$	decreased expression
$p_{33}$	impairing HDL-C
$p_{34}$	Tumor Necrosis Factor alpha (TNF-α) presence
$p_{35}$	oxidized HDL
$p_{36}$	Reconstituted HDL (rHDL)
$p_{37}$	exported cholesterol
$p_{38}$	inflammation condition
$p_{39}$	5A peptide
$p_{40}$	vitamin C and E

**Table 2:** List of transitions.

Transition	Biological Meaning	Transition	Biological Meaning
$t_{0}$	ApoA-I gene expression in the intestine	$t_{34}$	fatty acid release from HDL3
$t_{1}$	ApoA-I gene expression in the liver	$t_{35}$	fatty acids absorption
$t_{2}$	ApoA-I synthesis in the liver	$t_{36}$	absorbed FA in peripheral tissue
$t_{3}$	ApoA-I synthesis in the intestine	$t_{37}$	phospholipids source
$t_{4}$	discoidal HDL formation	$t_{38}$	discoidal HDL formation
$t_{5}$	enzymatic reactions	$t_{39}$	inflammatory function suppression
$t_{6}$	cholesterol export to HDL2 and HDL3	$t_{40}$	decreased efflux
$t_{7}$	cholesterol export to HDL2	$t_{41}$	increased exchange of TGs for cholesteryl esters from HDL3
$t_{8}$	enzymatic reactions	$t_{42}$	increased exchange of TGs for cholesteryl esters from HDL2
$t_{9}$	LCAT source	$t_{43}$	decreased concentration of HDL
$t_{10}$	cholesteryl ester transfer from HDL2	$t_{44}$	adiponectin decreased synthesis
$t_{11}$	cholesteryl ester transfer from HDL3	$t_{45}$	TNF-α removal
$t_{12}$	CETP source	$t_{46}$	inflammation source
$t_{13}$	formation of VLDL remnants	$t_{47}$	reduced LCAT-mediated cholesterol esterification
$t_{14}$	loss of fatty acids	$t_{48}$	reduced esterification removal
$t_{15}$	cholesteryl ester transfer from VLDL to HDL3	$t_{49}$	HDL3 hydrolysis
$t_{16}$	cholesteryl ester transfer from VLDL to HDL2	$t_{50}$	HDL2 hydrolysis
$t_{17}$	HDL catabolism	$t_{51}$	hydrolyzed HDL
$t_{18}$	catabolized HDL	$t_{52}$	retinoic acid receptor-alpha alterations
$t_{19}$	cholesterol binding	$t_{53}$	retinoic acid receptor-alpha alterations removal
$t_{20}$	cholesterol excretion	$t_{54}$	decreased expression induced by Interferon gamma (IFN-γ)
$t_{21}$	excreted cholesterol	$t_{55}$	IFN-γ removal
$t_{22}$	HDL2 remodeling	$t_{56}$	oxidized HDL-associated activity decrease
$t_{23}$	cholesterol export	$t_{57}$	oxidized High-Density Lipoprotein (oxHDL) removal
$t_{24}$	PLTP source	$t_{58}$	cholesterol source
$t_{25}$	HL source	$t_{59}$	HDL infusion
$t_{26}$	EL source	$t_{60}$	cholesterol export promoted by rHDL in ABCA1
$t_{27}$	Lipoprotein(a) forming	$t_{61}$	cholesterol export promoted by rHDL in SR-B1
$t_{28}$	LDL binding	$t_{62}$	cholesterol export promoted by rHDL in ABCG1
$t_{29}$	Lp(a) metabolism	$t_{63}$	inflammation state
$t_{30}$	cholesterol transfer	$t_{64}$	5A source
$t_{31}$	fatty acid release from IDL	$t_{65}$	inhibition of inflammation
$t_{32}$	fatty acid release from VLDL	$t_{66}$	treatment source
$t_{33}$	fatty acid release from HDL2	$t_{67}$	reduction of oxidative stress and restoration of redox balance

**Table 3:** Key transitions identified by in silico knock-out analysis in inflammatory and therapeutic HDL Petri net models. Table entries summarize dominant qualitative patterns observed in comparative knock-out simulations. Although different transitions emerged as the most influential in the inflammatory and therapeutic model variants, the regulation of inflammation and redox balance appeared as recurring biological themes that influenced network organization across both contexts.

Model Context	Transition ID	Biological Process Represented	Observed System-Level Impact (Summary)
Inflammation model (no therapy)	$t_{44}$	Adiponectin decreased synthesis	Strong redistribution of LDL/HDL remodeling fluxes; increased inflammatory load and impaired HDL-C formation
	$t_{56}$	Activity decrease (oxidized HDL-related)	Marked shift toward oxidized HDL and compensatory activation of alternative efflux pathways
	$t_{47}$	Reduced LCAT-mediated cholesterol esterification	Impaired maturation of HDL particles and increased inflammatory signaling
Therapeutic model (anti-inflammatory, antioxidant and rHDL-mediated restoration)	$t_{65}$	Inhibition of inflammation	Global suppression of inflammatory token accumulation and partial normalization of HDL functionality
	$t_{67}$	Reduction of oxidative stress and restoration of redox balance	Strong reduction of oxidized HDL burden and restoration of antioxidant-related processes
	$t_{60}$	Cholesterol export promoted by rHDL (ABCA1-dependent)	Enhanced cholesterol efflux toward rHDL and reduced intracellular cholesterol accumulation
	$t_{61}$	Cholesterol export promoted by rHDL (SR-B1-dependent)	Increased hepatic and renal cholesterol handling via SR-B1-mediated pathways
	$t_{62}$	Cholesterol export promoted by rHDL (ABCG1-dependent)	Reinforcement of peripheral cholesterol efflux and HDL remodeling

**Table 4:** Regulatory bottlenecks of HDL metabolism: mechanistic roles and clinical implications.

Bottleneck Transition	Core Biological Function	System-Level Role	Clinical/Translational Implication
$t_{47}$ (Reduced LCAT-mediated cholesterol esterification)	Conversion of free cholesterol into cholesteryl esters; HDL maturation	Metabolic choke point controlling structural maturation of HDL particles	Impaired LCAT activity may explain dysfunctional HDL and ineffective reverse cholesterol transport despite normal HDL levels.
$t_{56}$ (Oxidized HDL/activity decrease)	Oxidative modification of HDL and inhibition of transporter activity (ABCA1/ABCG1)	Inflammation-oxidation hub amplifying network instability and compensatory responses	Supports the role of oxidative stress as a driver of HDL dysfunction and a potential target for redox-based therapies.
$t_{44}$ (Adiponectin decreased synthesis)	Regulation of lipid metabolism and anti-inflammatory signaling	Integrator of metabolic and inflammatory signaling	Links adipose tissue dysfunction with HDL impairment; highlights the relevance of metabolic syndrome in HDL biology.
$t_{65}$ (Inflammation inhibition)	Suppression of pro-inflammatory signaling pathways	Global master regulator restoring network stability	Suggests anti-inflammatory strategies as key drivers of HDL functional recovery.
$t_{67}$ (Oxidation reduction)	Reduction of oxidative stress and restoration of redox balance	Shared regulatory framework model contexts	Identifies redox modulation as a central therapeutic axis in HDL-related disorders.
$t_{60}$–$t_{62}$ (rHDL-mediated cholesterol export)	Enhanced cholesterol efflux via ABCA1, SR-B1, and ABCG1 pathways	Effector layer restoring lipid flux	Explains why direct HDL infusion strategies may work only when upstream regulation is restored.

**Table 5:** Modifications introduced to the HDL metabolism model to represent the inflammatory state.

Place/Transition Name; Modification	Literature Support
inflammatory function suppression; increase in weights of arcs: ABCA1 ($p_{3}$) → discoidal HDL formation ($t_{38}$) (new weight: 3) ABCA1 ($p_{3}$) → enzymatic reactions ($t_{5}$) (new weight: 3)	Adipose tissue inflammation impairs the expression and activity of cholesterol transporters such as ABCA1, reducing cholesterol efflux from adipocytes. Since ABCA1 is a key mediator of both cholesterol transfer and the generation of nascent HDL particles, its dysfunction promotes the production of immature HDL with diminished capacity for reverse cholesterol transport [15,41,42].
weight 3 on cholesterol export ($t_{23}$) to ABCG1 ($p_{5}$) arc	Loss of adipocyte ABCA1 has been associated with a compensatory increase in ABCG1 expression, indicating that cells enhance alternative cholesterol export pathways to preserve intracellular cholesterol homeostasis [43].
Added new place—TG-enriched HDL-C clearance ($p_{26}$)	Elevated CETP activity promotes the exchange of triglycerides for cholesteryl esters between lipoproteins, generating HDL particles enriched in triglycerides. These modified HDL particles exhibit reduced stability and are cleared more rapidly from the circulation, contributing to lower circulating HDL-C concentrations [15,44,45].
adiponectin decreased production; increase in weights of arcs: ABCA1 ($p_{3}$) → discoidal HDL formation ($t_{38}$) (new weight: 5) ABCA1 ($p_{3}$) → enzymatic reactions ($t_{5}$) (new weight: 5) ABCA1 ($p_{3}$) → absorbed FA in peripheral tissue ($t_{36}$) (new weight: 3) lipid-poor ApoA-I involved in HDL biogenesis ($p_{2}$) → discoidal HDL formation ($t_{4}$) (new weight: 3) lipid-poor ApoA-I involved in HDL biogenesis ($p_{2}$) → discoidal HDL formation ($t_{38}$) (new weight: 3)	Reduced adiponectin levels have been associated with impaired HDL metabolism and disrupted cholesterol homeostasis. Experimental studies in animal models have shown that adiponectin deficiency lowers the expression of both ApoA-I and ABCA1, leading to reduced ApoA-I availability and diminished HDL-C formation, although comparable evidence has not been consistently demonstrated in humans [15,46].
reduced LCAT-mediated cholesterol esterification; increase in weights of arcs: LCAT ($p_{9}$) → enzymatic reactions ($t_{5}$) (new weight: 3) LCAT ($p_{9}$) → enzymatic reactions ($t_{8}$) (new weight: 3)	The association of serum amyloid A (SAA) with HDL particles during chronic inflammation disrupts normal HDL remodeling by reducing the efficiency of LCAT-mediated cholesterol esterification. As a consequence, the conversion of nascent HDL into mature HDL particles is impaired, leading to defective HDL maturation and reduced reverse cholesterol transport [3,6,7,15,44].
Added new place—HDL hydrolysis increased by sPLA2 ($p_{29}$)	Inflammatory conditions promote the release of sPLA2, which enhances the hydrolysis of phospholipids within HDL particles. This accelerates HDL particle degradation and contributes to a reduction in circulating HDL-C levels [15].
decreased expression; increase in weights of arcs: ABCA1 ($p_{3}$) → discoidal HDL formation ($t_{38}$) (new weight: 7) ABCA1 ($p_{3}$) → enzymatic reactions ($t_{5}$) (new weight: 7) ABCA1 ($p_{3}$) → absorbed FA in peripheral tissue ($t_{36}$) (new weight: 5) SR-B1 ($p_{6}$) → cholesterol export ($t_{6}$) (new weight: 3) ABCG1 ($p_{5}$) → cholesterol export ($t_{7}$) (new weight: 3)	Low-grade systemic inflammation impairs reverse cholesterol transport by suppressing the expression of key cholesterol transporters. Inflammatory signaling reduces retinoic acid receptor-α activity, leading to decreased expression of ABCA1, ABCG1, and SR-B1 in macrophages, thereby limiting cholesterol efflux and compromising HDL function [3,10,11,15].
decreased expression by IFN-γ; increase in the weights of arc: SR-B1 ($p_{6}$) → cholesterol export ($t_{6}$) (new weight: 5)	Interferon-γ has been shown to impair macrophage cholesterol handling by reducing the expression of SR-B1 while promoting macrophage activation. The resulting decrease in SR-B1 expression limits cholesterol efflux and contributes to impaired reverse cholesterol transport under inflammatory conditions [15].
activity decrease; increase in weights of arcs: ABCA1 ($p_{3}$) → discoidal HDL formation ($t_{38}$) (new weight: 9) ABCA1 ($p_{3}$) → enzymatic reactions ($t_{5}$) (new weight: 9) ABCA1 ($p_{3}$) → absorbed FA in peripheral tissue ($t_{36}$) (new weight: 7) ABCG1 ($p_{5}$) → cholesterol export ($t_{7}$) (new weight: 5)	Oxidized HDL particles amplify inflammatory signaling through activation of the NLRP3 inflammasome, promoting the release of pro-inflammatory mediators such as IL-1β and IL-18. This inflammatory response is accompanied by reduced activity of the cholesterol transporters ABCA1 and ABCG1, further impairing cholesterol efflux and HDL functionality [15,19,20,22].

**Table 6:** Modifications introduced to the model to represent therapeutic interventions.

Place/Transition Name; Modification	Literature Support
increase in weights of arcs: cholesterol transfer ($t_{30}$) → ABCA1 ($p_{3}$) (new weight: 3) cholesterol export ($t_{23}$) → ABCA1 ($p_{3}$) (new weight: 3)	The HDL-modulating effect of theobromine has been attributed to enhanced ABCA1 activity. This mechanism is proposed to involve phosphodiesterase inhibition, which alters intracellular cAMP signaling and promotes ABCA1-mediated cholesterol efflux, thereby supporting HDL biogenesis [5].
vitamins C and E action; increase in weights of arcs: ABCA1 ($p_{3}$) → oxidized HDL-associated activity decrease ($t_{56}$) (new weight: 3) ABCG1 ($p_{5}$) → oxidized HDL-associated activity decrease ($t_{56}$) (new weight: 3)	Supplementation with vitamins C and E has been shown to enhance the antioxidant properties of HDL by promoting beneficial changes in its protein composition, including increased levels of paraoxonase 1 (PON1). Since PON1 is a major determinant of HDL antioxidant capacity, its increased abundance improves protection against HDL oxidation, whereas PON1 deficiency is associated with diminished antioxidant activity [5].
niacin-increase in weight of arc spherical HDL2 ($p_{8}$) → HDL catabolism ($t_{17}$) to 3	The interaction between niacin and HDL catabolism is not explicitly discussed in the main text of the cited publication. The modification of this interaction in the Petri net model is therefore based on the conceptual pathway illustrated in Figure 3 rather than on direct evidence reported in the article [5].
increase in weights of arcs: fatty acid release ($t_{32}$) → fatty acid ($p_{21}$) (new weight: 3) ApoA-I synthesis ($t_{2}$, $t_{3}$) → lipid-poor ApoA-I involved in HDL biogenesis ($p_{2}$) (new weight: 3) cholesterol export ($t_{23}$) → ABCA1 ($p_{3}$) (increased weight by 2; new weight: 5) cholesterol transfer ($t_{30}$) → ABCA1 ($p_{3}$) (increased weight by 2; new weight: 5)	Activation of Peroxisome proliferator-activated receptor (PPAR) α enhances lipid metabolism by increasing the expression of lipoprotein lipase (LPL), thereby promoting triglyceride hydrolysis and fatty acid release. In addition, fibrates stimulate the synthesis of ApoA-I and upregulate ABCA1 expression, supporting HDL biogenesis and cholesterol efflux [5].
CETP inhibition; increase in weights of arcs: CETP ($p_{10}$) → cholesteryl ester transfer ($t_{10}$) (new weight: 3) CETP ($p_{10}$) → cholesteryl ester transfer ($t_{11}$) (new weight: 3)	CETP activity is inversely associated with circulating HDL-C concentrations because it mediates the exchange of cholesteryl esters and triglycerides between lipoproteins. Consequently, pharmacological inhibition of CETP has been extensively investigated as a strategy to reduce cholesteryl ester transfer and increase plasma HDL-C levels [5,9,10].
increased in weight of arc ApoA-I synthesis ($t_{2}$, $t_{3}$) to lipid-poor ApoA-I involved in HDL biogenesis ($p_{2}$) by 2; new weight: 5	Apabetalone (RVX-208), a bromodomain and extraterminal (BET) protein inhibitor, has been shown to enhance ApoA-I synthesis through epigenetic regulation. By increasing ApoA-I production, apabetalone promotes HDL biogenesis and favorably influences HDL-related metabolic pathways [5,10,11].
increase in weights of arcs: cholesterol export ($t_{23}$) to ABCA1 ($p_{3}$) (increased weight by 2; new weight: 7) cholesterol transfer ($t_{30}$) to ABCA1 ($p_{3}$) (increased weight by 2; new weight: 7) cholesterol export ($t_{23}$) to ABCG1 ($p_{5}$) (increased weight by 2; new weight: 5)	The ApoA-I mimetic peptide 5A has been shown to promote cholesterol efflux by activating the ABCA1- and ABCG1-mediated transport pathways. Enhanced cholesterol export through these transporters improves reverse cholesterol transport and has been associated with reduced atherosclerotic burden in preclinical studies [5].
Added new place-rHDL ($p_{36}$)	rHDL particles have been developed to enhance ABCA1-mediated cholesterol efflux from cholesterol-loaded macrophages. In addition to promoting reverse cholesterol transport, rHDL has been investigated as a therapeutic carrier for targeted delivery to macrophages and atherosclerotic plaques [27,28,47].
Added new arcs cholesterol export from SR-B1 ($p_{6}$) and ABCG1 ($p_{5}$) to rHDL ($p_{36}$)	Additional arcs connecting ABCG1 (*p*_5_) and SR-B1 (*p*_6_) with rHDL (*p*_36_) were introduced to represent potential transporter-mediated cholesterol efflux pathways supported by HDL mimetics and reconstituted HDL particles [27,28].
5A ($p_{39}$); changes: increased weight of every inflammation arc by 2 increased weight of every oxidation arc by 2 (new weight: 5)	The ApoA-I mimetic peptide 5A has been shown to attenuate both inflammatory responses and oxidative stress in experimental models, including human endothelial cells and rabbit carotid arteries. These findings indicate that, beyond enhancing cholesterol efflux, 5A exerts anti-inflammatory and antioxidant effects that contribute to its atheroprotective properties [5].

## Data Availability

The data supporting the findings of this study are available within the article and its Supplementary Materials. The Petri net-based model used in this study is provided in the Supplementary Materials. Additional information is available from the corresponding author upon reasonable request.

## Supplementary Materials

The following supporting information can be downloaded at https://www.mdpi.com/article/10.3390/ijms27188066/s1.
